# Influence of Body Composition on Gait Kinetics throughout Pregnancy and Postpartum Period

**DOI:** 10.1155/2016/3921536

**Published:** 2016-03-17

**Authors:** Marco Branco, Rita Santos-Rocha, Filomena Vieira, Maria-Raquel Silva, Liliana Aguiar, António P. Veloso

**Affiliations:** ^1^Neuromechanics of Human Movement Research Group, Interdisciplinary Centre for the Study of Human Performance (CIPER), Faculty of Human Kinetics, Estrada da Costa, 1499-002 Cruz Quebrada-Dafundo, Portugal; ^2^Polytechnic Institute of Santarém-Sport Sciences School of Rio Maior (IPS-ESDRM), Avenida Dr. Mário Soares 110, 2040-413 Rio Maior, Portugal; ^3^Faculty of Human Kinetics, University of Lisbon (UL-FMH), Estrada da Costa, 1499-002 Cruz Quebrada-Dafundo, Portugal; ^4^Faculty of Health Sciences, University Fernando Pessoa, Rua Carlos da Maia 296, 4200-150 Porto, Portugal; ^5^Research Centre for Anthropology and Health, University of Coimbra, Calçada Martim de Freitas, 3000-456 Coimbra, Portugal; ^6^Institute of Environmental Health, University of Lisbon (UL-FML), Avenida Professor Egas Moniz, 1649-028 Lisboa, Portugal

## Abstract

Pregnancy leads to several changes in body composition and morphology of women. It is not clear whether the biomechanical changes occurring in this period are due exclusively to body composition and size or to other physiological factors. The purpose was to quantify the morphology and body composition of women throughout pregnancy and in the postpartum period and identify the contribution of these parameters on the lower limb joints kinetic during gait. Eleven women were assessed longitudinally, regarding anthropometric, body composition, and kinetic parameters of gait. Body composition and body dimensions showed a significant increase during pregnancy and a decrease in the postpartum period. In the postpartum period, body composition was similar to the 1st trimester, except for triceps skinfold, total calf area, and body mass index, with higher results than at the beginning of pregnancy. Regression models were developed to predict women's internal loading through anthropometric variables. Four models include variables associated with the amount of fat; four models include variables related to overall body weight; three models include fat-free mass; one model includes the shape of the trunk as a predictor variable. Changes in maternal body composition and morphology largely determine kinetic dynamics of the joints in pregnant women.

## 1. Introduction

During pregnancy and in the postpartum period, the woman's body experiences large changes in morphology, physiology, and, consequently, body composition. The association between body composition, particularly the increase in maternal weight, and health related problems is known for mother and child well-being [1, 2]. According to the Institute of Medicine and National Research Council of the National Academies [3] women with a body mass index (BMI) lower than 19.8 kg/m^2^ (underweight) should increase their weight between 12.5 kg and 18 kg; women with BMI between 19.8 kg/m^2^ and 26.0 kg/m^2^ (normal weight) must have an increase in body weight between 11.5 kg and 16 kg; women with BMI between 26 kg/m^2^ and 29.0 kg/m^2^ (overweight) must have an increase in weight of 7 kg to 11.5 kg; women with BMI equal or greater than 29 kg/m^2^ (obese) should gain at least 6.8 kg. For women carrying twins, the recommended target total weight gain at term is 16.0 to 20.5 kg [3, 4].

Weight gain during pregnancy has been widely studied and is reported in several studies as described below. In general, during pregnancy the weight gain stands at around 11 kg [5–7] although it has been increasing in recent years from 9 kg [8] to 14.5 kg [9] in nonobese women, with much of these gains occurring during the 2nd trimester. However, in late pregnancy, women accumulate an average of 4 kg of body fat [5, 7, 8]. The prevalence of women with normal weight significantly decreased between the 2nd and the 3rd trimesters from 63.2% to 39.5%, respectively [10]. After delivery, the woman's weight remains above her prepregnancy body weight [5] or in early pregnancy [6].

Women classified as obese in early pregnancy have a significantly higher sum of skinfolds thickness and higher fat mass gains compared to normal weight women [6]. Although different studies showed an increase in the skinfolds thickness throughout pregnancy, changes were not always significant. The skinfolds that showed significant increases were subscapular, suprailiac, and thigh [11]; triceps, biceps, and subscapular [12]; and midthigh and calf [10].

Body dimensions of pregnant women measured by the segmental girths show that thigh [10, 11], hip, and calf girths [11] significantly increase during pregnancy. Also between the 2nd and the 3rd trimesters there is an increase of body fat areas of the midthigh and calf and the total fat mass [10].

The influence of body segment parameters in the estimation of inverse dynamics solutions has been investigated by some studies conducted in the last decade. Some of those studies compared the values of inverse dynamics computations using different methods for determination of the inertial characteristics through anthropometric data [13, 14], by the quality of the kinematic and dynamic inputs, and of the biomechanical model anatomical data [15]. Statistical differences were found between some conditions; however, only Jensen et al. [16] compared the inertial characteristic in pregnant women, finding differences only in lower trunk inertias.

No studies were found relative to the influence of body composition and morphological changes in kinetic parameters of gait, during pregnancy and the postpartum period, and it is not known to what extent these changes contribute to the internal load of women, on this special stage of life.

Thus, the purposes of this study were to quantify maternal anthropometric and body composition changes throughout pregnancy and in the postpartum period and to identify the contribution of these parameters on the lower limb joints kinetic during gait.

## 2. Materials and Methods

### 2.1. Subjects

The sample was composed of eleven healthy Caucasian women (33.2 ± 1.6 years, range 32–37) without musculoskeletal problems, neuromuscular disorders, or other diseases (Table 1).

All participants have volunteered to participate in the study through personal contacts in fitness clubs or health centers in Lisbon (Portugal) from January 2010 to May 2013. None of the participants had a contraindication for the practice of physical exercise. All subjects gave written informed consent before participation in the study. This study was approved by the Ethical Council of the Faculty of Human Kinetics, University of Lisbon, Portugal.

### 2.2. Data Collection and Processing

Data were collected at the Laboratory of Biomechanics and Functional Morphology of the Faculty of Human Kinetics in four periods: at a gestational age of 14.2 ± 2.4 weeks, at 27.3 ± 1.0 weeks, and at 36.3 ± 0.9 weeks, and in the postpartum period at 20.6 ± 5.2 weeks.

The anthropometric variables collected were weight, height, six skinfolds (subscapular, triceps, biceps, iliac crest, front thigh, and medial calf); four girths (abdominal, gluteal, midthigh, and calf); and three breadths (biiliocristal, thoracic, and biacromial). All anthropometric data were measured according to the International Society for the Advancement of Kinanthropometry (ISAK) standardized measurement protocol [17], with an exception for abdominal girth, which was measured 2 cm below the belly button (navel), and the thoracic breadth, which was measured at the level of the last rib. Data were collected by ISAK certified anthropometrists. Based on these measurements, other variables were calculated, including body weight gain in each trimester by reference to prepregnancy body weight (self-reported by pregnant in a specific questionnaire form); the body density and the percentage of fat mass [18], muscle, and fat areas of the thigh and lower leg [19]; the body fat and fat-free mass; and the biiliocristal-acromial and abdominal-gluteal ratio and BMI.

Kinematic and kinetic parameters were collected through 12 infrared high-speed cameras (Oqus-300, Qualisys, Sweden) at a rate of 200 Hz and three force platforms (two Kistler AG, Winterthur, Switzerland, and one AMTI, Advanced Mechanical Technology, Inc., Watertown), at a rate of 1000 Hz. Spherical reflective markers were placed on the skin in lower limb segments with double-sided adhesive tape at predefined locations according to recent recommendations [20]. Kinetic and kinematic data were synchronized to the same file through software Qualisys Track Manager (QTM; Qualisys AB, Gothenburg, Sweden). Data were collected during three nonconsecutive minutes' walking at a comfortable speed and were considered the last four cycles performed by each participant. The procedures were fully described in a previous paper [21, 22]. The kinetics parameters considered in the present study are referred to the right lower limb.

### 2.3. Statistical Analysis

The statistical procedures were conducted using IBM SPSS Statistics 22 software for Windows. Shapiro-Wilk normality tests were conducted and not assumed for all cases. Repeated measures ANOVA analysis was used to verify differences of body composition variables between groups. For variables and groups that do not commit all assumptions for repeated measures analysis, the Friedman test was performed. The enter method was used to develop the linear regression prediction models. Only one predictor variable can enter this technique because of the sample size [23].

## 3. Results

### 3.1. Anthropometric and Body Composition Profile

The anthropometric and body composition profiles of the women during pregnancy and in postpartum period are described in Table 2.

All anthropometric variables were significantly influenced by pregnancy, with an exception for midthigh girth and biiliocristal-biacromial ratio.

Although the biacromial breadth was significantly different between the 1st and the 3rd trimester and from the 1st trimester and postpartum period, the biiliocristal breadth was only significantly different from the 1st trimester and the 3rd trimester (an increase of 1.5 cm, *p* ≤ 0.001). Thus, no significant differences (*p* > 0.05) were observed for the biiliocristal-biacromial ratio (Table 2).

The thoracic breadth shows changes between all pairs of collection phases, with an exception for the 1st trimester and postpartum period, increasing 2.6 cm from the 1st to the 3rd trimester and decreasing the same value from late pregnancy to postpartum period.

The abdominal girth shows a mean increase throughout pregnancy of 16.5 cm and a significant decrease of 14.7 cm from 3rd trimester to postpartum period. The gluteal girth also shows significant increases of 3.1 cm and 4.3 cm from 1st to 2nd and 3rd trimesters and a significant decrease of 3.2 cm from late pregnancy to postpartum period. The calf girth only shows a significant increase of 1.1 cm from early to late pregnancy. Only midthigh girth has no changes in any of the phases observed.

The subscapular and biceps skinfolds show a significant increase of, respectively, 1.9 mm and 0.3 mm from 1st to 3rd trimester, without changes from 3rd trimester to postpartum period. The tricipital skinfold increases its size from 1st, 2nd, and 3rd trimester to postpartum period, respectively, 3.8 mm, 2.5 mm, and 3.7 mm. The iliac crest, front thigh, and calf skinfolds have no changes throughout pregnancy and postpartum period. The sum of the skinfolds shows a significant increase of 6.0 cm from 1st to 3rd trimester, without other changes throughout pregnancy and for postpartum period, and the relative value of fat mass shows no changes during pregnancy and in the postpartum period.

Body mass (*p* ≤ 0.01) and weight gain (*p* ≤ 0.02) showed a significant increase throughout pregnancy and a significant decrease from the 3rd trimester to postpartum period (*p* ≤ 0.01 and *p* ≤ 0.02, resp.; Table 2).

By the recommendations of the Subcommittee on Nutritional Status and Weight Gain During Pregnancy of the Committee on Nutritional Status During Pregnancy and Lactation, Institute of Medicine, our participants demonstrated a normal weight (BMI ≥ 19.8 to 26.0 kg/m^2^) during the first two trimesters and in the postpartum period. They showed overweight values in the last trimester (BMI > 26.0 to 29.0 kg/m^2^). On the other hand, pregnancy weight gain was lower than the recommendations during the first two trimesters, since participants weight gain should had varied from 11.5 to 16 kg as their BMI was normal in the 1st and the 2nd trimesters. In contrast, during the 3rd trimester, the mean body weight gain was above the recommendations [3].

Only the segmental areas of the calf were affected by pregnancy. The muscular area of the calf had a significant increase of 4.2 cm^2^ from the 1st to the 3rd trimester and a significant decrease of 3.9 cm^2^ from the 3rd trimester to the postpartum period. The fat area of the calf also increased significantly from the 1st to the 3rd trimester and for the postpartum period, respectively, of 2.4 cm^2^ and 2.6 cm^2^. The paired comparisons of the total area of the thigh significantly increased 14.5 cm^2^ and 16.4 cm^2^, respectively, from the 1st to the 2nd and 3rd trimesters. The muscular and fat areas of the thigh did not show any changes throughout pregnancy and postpartum period.

As excepted, women's fat mass and fat-free mass weight were influenced by the stage of pregnancy or postpartum period. Body fat increased in the 1st to the 2nd and the 3rd trimesters, respectively, 2.9 kg and 3.7 kg, decreasing by the end of pregnancy to postpartum period in 2.1 kg. The fat-free mass was significantly increased by 6.1 kg between the 1st and the 2nd trimester and by 3.5 kg through the end of the pregnancy. After delivery, this variable showed a significant decrease when compared to the values of the 2nd and 3rd trimesters, in 3 kg and 6.6 kg, respectively.

The abdominal-gluteal ratio shows changes throughout pregnancy and in the postpartum period. No changes were found from the 1st trimester until postpartum period. No significant changes were observed in the biiliocristal-biacromial ratio during this study; however, this variable was used in calculating women's acromion-iliac ratio. In every collection phase, acromion-iliac ratio showed greater values than 0.76, which means that, during pregnancy and for postpartum period, women keep a trunk with a rectangular shape [24]. BMI increased 3.7 kg/m^2^ from the 1st to the 3rd trimester and decreased 3.1 kg/m^2^ from the late pregnancy to the postpartum period.

The joints moment and power used for the calculation of the regression models are shown in Table 3.

The kinetic variables under study are described more deeply in a previous paper [25]. However, those whose changes were identified as affected by the stage of pregnancy were considered for this study. Regarding the ground reaction forces (GRF) only the vertical component of the 3rd peak showed the influence of the stage of pregnancy or postpartum period. In the joint moments were considered the 2nd peak of the ankle and the 1st and 2nd peak of the hip in the sagittal plane and the 1st peak of the hip joint in the transverse plane. For the joint powers were considered the 2nd peak of the knee and hip joints in the sagittal plane and the 1st peak of the hip joint in the transverse plane.

### 3.2. Regression Models for Joint Moments

The building of predictive models for the kinetic parameters through anthropometric variables can provide additional information about the dynamics of the internal load in pregnant women. The relationships between anthropometric variables and the knee and hip joints moment in the sagittal plane were found in three cases, which are given in Table 4.

**Figure 1:**
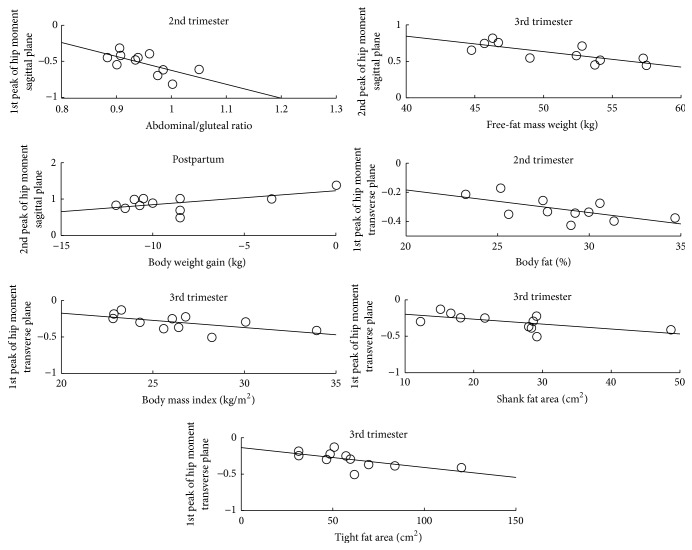
Scatter plots and regression lines of the joint moment (M·m/kg) predictive models.

The relation between anthropometric variables and the joint moments peaks are represented in Figure 1.

The abdominal-gluteal girth ratio is a significant predictor (*p* < 0.03) of the 1st peak of the hip joint moment in women's 2nd trimester of pregnancy and explains 38% of its variability. In the 3rd trimester, 53.4% of the variability of the 2nd peak of the hip joint moment can be significantly explained by the fat-free mass weight (*p* < 0.01). In the postpartum period, the body weight gain is a significant predictor (*p* < 0.05) of the 2nd peak of the hip joint moment and explains 29.6% of its variability.

In Table 4 are the regression models to predict joint moments in the transverse plane. The hip joint moments in 2nd trimester are significantly predicted by percentage of body fat (*p* < 0.05). This model is significant and explains 33.3% of the variability of the hip joint moment.

The hip joint moments for 3rd trimester are significantly predicted by body mass index (*p* < 0.05), by calf fat area (*p* < 0.05), and by thigh fat area (*p* < 0.05). These models explain 29.6%, 31.6%, and 32.4% of the variability of the hip joint moments in transverse plane, respectively.

### 3.3. Regression Models for Joint Powers

The performed analysis found several regression models that allow predicting the joint powers for the four phases of body composition variables. In the sagittal plane only the knee and hip present prediction models regarding the four phases studied (Table 4).

**Figure 2:**
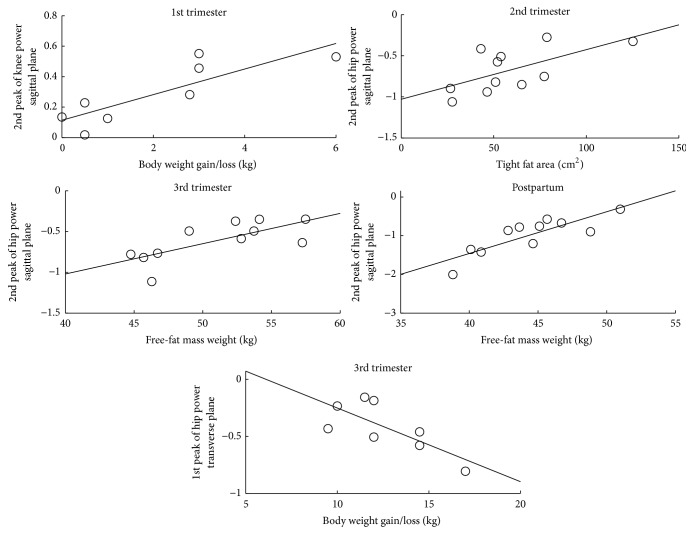
Scatter plots and regression lines of joint power (W/kg) predictive models.

The relation between anthropometric variables and the joint power peaks are represented in Figure 2.

In the 1st trimester, the power of the knee joint is significantly predicted in 65% of its variability by body weight gain (*p* ≤ 0.01). In the 2nd trimester, thigh fat area is a significant predictor of the hip joint power, which explains 32.8% of the total variability. The fat-free mass weight is a significant predictor of the hip joint power in the 3rd trimester and the postpartum period, explaining 46.8% and 66.9% of the total variability of the model, respectively.

In the transverse plane, the analysis has found regression models only for the hip joint, for the 3rd trimester of pregnancy (Table 4).

Body weight gain is a significant predictor (*p* < 0.05) of hip joint power in the transverse plane. The model explains 47.3% of its total variability.

Although the ground reaction forces (GRF) are the loading response of the body mass in the ground, it has not been defined how much of this response is due to the anthropometric or body composition variables. In Table 4 are presented the regression model for GRF with an anthropometric predictor.

**Figure 3:**
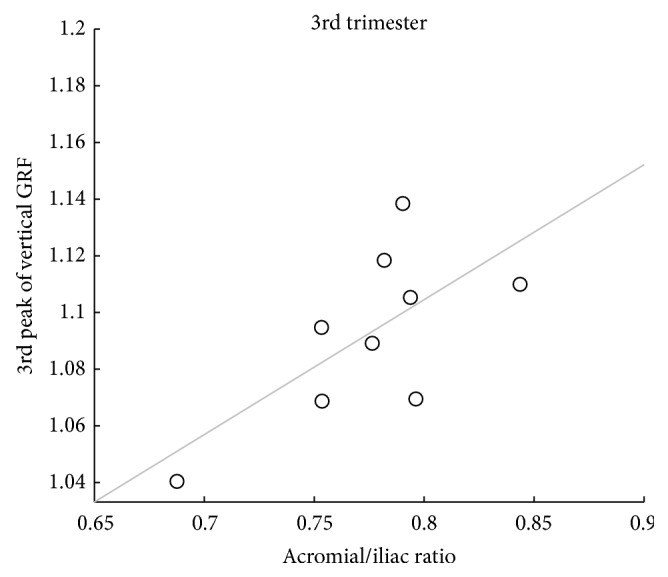
Scatter plots and regression lines of ground reaction force (in the percentage of body weight) predictive models.

The relation between anthropometric variables and GRF peaks is represented in Figure 3.

The biiliocristal-biacromial ratio is a significant predictor (*p* < 0.05) of the 3rd peak of the vertical GRF, when the pregnant women are in the 3rd trimester of pregnancy, and explains 38.4% of its variability.

## 4. Discussion

This study's first objective was to assess and quantify women's anthropometric and body composition changes during pregnancy and in the postpartum period. Despite the small size of the sample, this was a longitudinal study that is in agreement with other longitudinal studies conducted during pregnancy [26, 27]. In general, body composition variables showed an increase throughout pregnancy and a reduction from late pregnancy to the postpartum period. Nevertheless, most of these variables did not differ between the 1st trimester and postpartum period, with an exception for body weight loss, which is significantly lower after delivery than in the 1st trimester (*p* = 0.012).

During pregnancy, several physiological modifications occur in the pregnant woman's body to guarantee mother's energy needs and health and to support fetus growth and development [4, 26, 28]. In fact, maternal body composition has been one of the most studied, but there is lack of studies about kinetics parameters of gait during pregnancy.

In our study, both weight gain and the body mass index were affected by pregnancy with significant increases during pregnancy [27] and significant decreases in the postpartum period, as in recent studies [29]. However, that variation in weight gain was lower than the recommendations during the first two trimesters and higher in the 3rd trimester [3, 4, 27]. It has been highlighted that maternal fat gains are associated with important implications for maternal and offspring health and with a greater postpartum fat retention in the mother [27]. Also, the maternal body weight gain may also interfere with important kinetics parameters of gait as observed in our results of gait's kinetics parameters. The study of the influence of morphology on the internal load of women during pregnancy and in the postpartum period has not been previously studied.

In another recent study [26] to determine women's metabolic profile, twenty-one healthy women were evaluated: eleven at preconception, during pregnancy, and one year postpartum, and ten had no interval pregnancy so were assessed at baseline and a one-year interval. Weight, fat-free mass (kg), fat mass (kg), and the percentage of body fat were assessed by hydrodensitometry. Significant differences (*p* < 0.05) were observed for weight and fat-free mass from preconception (59.7 (53.5–85.6) kg and 42.5 (39.0–49.2) kg, resp.) to pregnancy (77.2 (62.9–94.3) kg and 54.7 (46.7–57.5) kg, resp.). In contrast, no significant differences were observed in preconception versus postpartum period for these two variables. Also, no significant differences (*p* > 0.05) were observed in body fat according to body size and in the percentage of body fat between preconception (20.7 (13.7–37.4) kg and 30.0 (24.1–43.8)%) and pregnancy (24.2 (14.9–38.7) kg and 30.6 (24.8–40.2)%) and between pregnancy and postpartum periods (18.4 (13.8–41.3) kg and 29.3 (25.2–46.2)%). Similar results to ours in the postpartum period were observed for fat-free mass (44.3 (38.8–49.6) kg), body fat according to body size (18.4 (13.8–41.3) kg), and the percentage of body fat (29.3 (25.2–46.2)%).

The biiliocristal breadth has a significant increase of 1.5 cm between the 1st and the 3rd trimester and although there is a reduction in late pregnancy to the postpartum period, this is not significant, which may suggest that the distance between the iliac bones remains after pregnancy.

As expected, abdominal and the gluteal girths suffered major changes throughout pregnancy. However, these variables recovered in the postpartum period to values similar to those found in early pregnancy. The calf girth increased 1.1 cm from the 1st to the 3rd trimester as Pérez et al. [10] found. These authors also found changes in the midthigh girth, which was not demonstrated in this study.

Although the sum of skinfolds showed a significant increase between the 1st and 3rd trimester of pregnancy, mainly caused by subscapular, triceps, and biceps skinfolds, only the triceps skinfold presented a significant increase of almost 4 mm throughout pregnancy to the postpartum period. Although no significant changes were observed in the fat mass percentage, the accumulation of fat mass seems to happen mostly in the upper region of the trunk and upper limbs, keeping or increasing their thickness in the postpartum period. Also, in relation to fat mass weight, significant increases from the 1st to the 2nd trimester and from the 2nd to the 3rd trimester of pregnancy were showed. After delivery, the fat mass weight decreases, showing a recovery to levels similar to the first stage of pregnancy. In the same way, the fat-free mass weight increases during pregnancy, which may be related to the natural increase in the tissues and fluids and also the weight of the fetus. It decreases again in the postpartum period.

The bicompartmental assessment of body composition of the lower limbs can be observed by the total, muscle, and fat areas of the thigh and calf. Pérez et al. [10] have found increases in the thigh fat area and further in calf fat area, between the 2nd and the 3rd trimester. In this study, only the total area of the thigh showed significant changes, increasing 14.5 cm^2^ and 16.4 cm^2^, respectively, from the 1st to the 2nd and from the 2nd to the 3rd trimesters of pregnancy. In the calf segment, significant increases were found throughout pregnancy, for both fat and muscle areas, but only the last variable decreases in the postpartum period.

The abdominal-gluteal ratio shows the volume distribution between the lower torso and the pelvic region. Throughout pregnancy, it changes so that in late pregnancy the distribution of the volume of the abdomen (mainly anterior) and the volume of the hips (mainly posterior) is the same. The biiliocristal-biacromial ratio does not show significant changes, meaning that pregnant women keep a rectangular trunk shape [24].

In the 3rd trimester, the regression models to predict relative GRF of women show that higher ratio between iliocristal and acromial breadth has an increased magnitude of the 3rd peak of vertical GRF (Figure 3).

After application of the linear regression technique for predicting women's internal loading during pregnancy and the postpartum period through anthropometric variables, twelve models were developed. Four of these models include independent variables associated with the amount of fat; four models include independent variables related to overall body weight; three models include fat-free mass as a predictor variable; and a model includes the shape of the trunk as a predictor variable. A summary of the interaction between the anthropometric variables and the joint kinetic variables of the lower limb, in the four studied stages, is represented in Figures 1 and 2.

In the 1st trimester of pregnancy, a higher body weight gain leads to an increased production of mechanical energy of the knee extensors during the midstance phase. This can be interpreted as a normal muscle response to the increase of the body weight of the woman to hold the body support function. However, in situations where the woman is standing or walking for a longer time, the possibility to complain of fatigue, discomfort, and pain in the lower limbs is greater. Moreover, these results are also meant to show the possibility to control an overload of the musculoskeletal system, reducing the increase in body mass. Noting that such information may not overlap the weight increase associated with the proper developing fetus.

In the 2nd trimester, the regression models allow predicting that a greater abdominal-gluteal ratio and a higher percentage of body fat have an increased participation of the extensors and external rotators of the hip, respectively, during the loading response phase. Pointing that the support of the body is associated with the body composition and to the morphology of the woman, we emphasize once again that the weight transfer between limbs and the stabilization of the body can be better controlled by a lower relative amount of fat. Also, in this stage of pregnancy, greater thigh fat area leads to a decreased ability to perform the eccentric contraction of the hip flexors during the terminal stance phase, revealing less control in advance and descent of the body towards the contact with the ground.

In the 3rd trimester, a higher body mass index and larger areas of fat of the calf and the thigh increased the participation of the hip external rotators during the loading response phase. Also, a higher body weight gain, until this trimester, induces to a greater eccentric contraction of the hip external rotators also in this phase of the walking cycle, associating the increase of these variables with a greater need to control the deceleration of the pelvis. Conversely, a higher fat-free mass leads to a lower need for stabilizing the trunk, which is associated with a lower eccentric contraction of the hip flexors during the terminal stance and a lower participation of the hip flexors during the preswing phase.

A higher weight of fat-free mass and a greater loss of body mass, in the postpartum period, leads, respectively, to a lower absorption of mechanical energy of the hip flexors during terminal stance phase and to a lower participation of the same muscles during the preswing phase, showing that there is an opposite effect to what were found in pregnancy and that the achievement of the gait cycle is mechanically more efficient with more fat-free mass.

Considering the limitations on the use of simple linear regression techniques, it should be appreciated that the variability in the explanation of the models has relatively high levels in five of them, whose values are very close to or above 50%. The highest levels of explanation in the models variability occurred in the 1st trimester and the postpartum period, during the phases of the gait cycle, in which there is an increased support of the body in one lower limb. The reason for this to happen in the early period of pregnancy and the postpartum period may show that the neuromuscular system of the woman is not prepared for morphological changes happening in her body. This information is relevant not only for the pregnant women but also for health and physical exercise professionals [4], mainly because variables like fat-free mass and loss or weight gain can be relatively controlled and associated with physical activity.

## 5. Conclusions

This study showed significant changes during pregnancy, regarding the variables associated with global body composition. The absolute amount of fat increased significantly to the end of pregnancy, especially by the contribution of the adipose tissue accumulated in the skinfolds of the upper limbs and upper trunk. Regarding the relative values of fat, there were no changes during pregnancy and to the postpartum period.

The shape of the trunk in a frontal perspective, the fat-free mass, and the weight gain/loss appeared as major predictors of the joint kinetics of women throughout pregnancy and in the postpartum period. Most of the variables related to the amount of fat are predictors of the muscles involved in motor actions of the transverse plane. Most of the developed models were used to predict muscle participation involving the hip joint in the sagittal and transverse planes of motion, emphasizing that this joint is the one that is under greater influence of anthropometric variables.

The body weight gain/loss was the variable that most explained the mechanical energy of the lower limb joints, showing that they had the most important role in the muscle contractions' dynamics, especially in the 1st and 3rd trimesters of pregnancy.

The morphological changes that occur during pregnancy had a greater influence on internal load involving the hip.

Although, due to the sample size, regression models had only one predictor variable, the explanation indices of the models (adjusted *R*-squared) were quite high, indicating that body composition and pregnant morphology change during pregnancy, largely determining the kinetic dynamics of the female joints in this particular stage of life.

Monitoring maternal body composition during pregnancy and the kinetic parameters of gait is a complex process. However, this process would be of great importance, especially in the 3rd trimester, if included in the health care procedure during pregnancy.

It is recommended for further studies the increase of the sample to allow regression models with more variables, including not only the body composition parameters but also physical activity before and during pregnancy.

## Figures and Tables

**Table 1:** Weight, body mass index (BMI), and gestational weeks of the participants (*N* = 11) before, during, and after pregnancy.

Variables	Before pregnancy	1st trimester	2nd trimester	3rd trimester	Postpartum
Height (m)	—	1.64 ± 0.04	—	—	—
Weight (kg)	60 ± 7.1	61.1 ± 6.6	66.6 ± 8.5	71.0 ± 8.0	62.4 ± 7.4
BMI (kg/m^2^)	22.5 ± 3.1	22.7 ± 2.8	24.7 ± 3.6	26.4 ± 3.4	23.2 ± 3.3
Weeks of gestation	—	14.2 ± 2.4	27.3 ± 1.0	36.3 ± 0.9	20.6 ± 5.2

**Table 2:** Anthropometric and body composition variables of the participants during pregnancy and in the postpartum period (*N* = 11). Mean values ± standard deviation of variables and significant values of post hoc tests (*p* < 0.05).

	1st trimester	2nd trimester	3rd trimester	Postpartum	Sig.
Anthropometry					
Biacromial breadth (cm)	36.5 ± 1.9	36.5 ± 0.9	37.1 ± 1.1	36.6 ± 1.8	^(b)^0.038
					^(d)^0.025
Biiliocristal breadth (cm)	27.6 ± 2.0	28.2 ± 2.0	29.0 ± 2.4	27.9 ± 1.8	^(b)^0.001
Thoracic breadth (cm)	24.1 ± 1.6	25.6 ± 1.1	26.7 ± 1.2	24.1 ± 1.6	^(a)^0.022
					^(b)^0.004
					^(c)^0.016
					^(e)^0.009
					^(f)^0.002
Abdominal girth (cm)	87.1 ± 6.7	96.8 ± 6.6	103.6 ± 7.2	88.9 ± 6.5	^(a)^ <0.001
					^(b)^ <0.001
					^(c)^ <0.001
					^(e)^ <0.001
					^(f)^ <0.001
Gluteal girth (cm)	99.0 ± 7.2	102.1 ± 7.5	103.3 ± 7.3	100.1 ± 7.7	^(a)^0.006
					^(b)^0.003
					^(f)^0.028
Midthigh girth (cm)	51.4 ± 5.4	53.1 ± 5.3	53.3 ± 5.1	52.6 ± 4.6	ns
Calf girth (cm)	35.8 ± 2.3	36.5 ± 2.8	36.9 ± 3.0	36.3 ± 2.7	^(b)^0.044
Biiliocristal-biacromial ratio	0.76 ± 0.05	0.77 ± 0.04	0.78 ± 0.04	0.76 ± 0.04	ns
Abdominal-gluteal ratio	0.88 ± 0.05	0.95 ± 0.05	1.0 ± 0.06	0.89 ± 0.03	^(a)^ <0.001
					^(b)^ <0.001
					^(c)^ <0.001
					^(e)^ <0.001
					^(f)^ <0.001

Body composition					
Body mass (kg)	61.1 ± 6.6	66.6 ± 8.5	71.0 ± 8.0	62.4 ± 7.4	^(a)^0.003
					^(b)^0.003
					^(c)^0.003
					^(e)^0.014
					^(f)^0.005
Body weight gain (kg)	2.1 ± 2.0	7.9 ± 3.0	12.6 ± 2.5	−8.6 ± 3.7	^(a)^0.012
					^(b)^0.012
					^(c)^0.011
					^(d)^0.012
					^(e)^0.012
					^(f)^0.011
Subscapular skinfold (mm)	11.5 ± 4.4	12.6 ± 4.4	13.3 ± 4.1	13.4 ± 6.5	^(a)^0.006
Triceps skinfold (mm)	16.2 ± 3.9	17.5 ± 4.2	16.3 ± 4.4	20.0 ± 5.3	^(d)^0.015
					^(e)^0.020
					^(f)^0.030
Biceps skinfold (mm)	6.8 ± 2.6	6.7 ± 3.1	7.1 ± 2.9	7.6 ± 3.5	^(b)^0.028
Iliac crest skinfold (mm)	19.1 ± 4.0	20.7 ± 4.9	19.0 ± 5.1	17.4 ± 4.3	ns
Front thigh skinfold (mm)	20.4 ± 7.5	23.4 ± 9.4	23.8 ± 8.1	23.4 ± 6.0	ns
Medial calf skinfold (mm)	13.4 ± 5.7	14.1 ± 6.5	14.3 ± 5.0	14.7 ± 5.7	ns
Skinfolds sum (mm)	124.7 ± 24.5	131.5 ± 30.3	130.7 ± 27.7	132.8 ± 27.9	^(b)^0.030
Fat mass (%)	26.4 ± 5.2	28.6 ± 3.2	28.1 ± 3.6	28.6 ± 4.0	ns
Fat mass weight (kg)	16.3 ± 4.5	19.2 ± 4.5	20.1 ± 4.4	18.0 ± 4.5	^(a)^0.038
					^(b)^0.022
Fat-free mass weight (kg)	44.8 ± 3.5	47.4 ± 4.4	50.9 ± 4.6	44.4 ± 3.7	ns
Thigh area (cm^2^)	211.9 ± 45.8	226.4 ± 46.4	228.3 ± 44.9	221.9 ± 38.8	ns
Thigh muscle area (cm^2^)	161.8 ± 37.3	167.6 ± 26.8	168.2 ± 24.8	164.1 ± 27.8	ns
Thigh fat area (cm^2^)	50.2 ± 20.0	58.8 ± 27.8	60.1 ± 25.2	57.8 ± 17.2	^(b)^0.041
					^(f)^0.047
Calf area (cm^2^)	102.6 ± 13.3	106.6 ± 17.0	109.2 ± 18.0	105.6 ± 15.6	^(b)^0.016
					^(d)^0.026
Calf muscle area (cm^2^)	79.9 ± 9.0	82.0 ± 9.4	84.1 ± 11.4	80.2 ± 9.0	^(a)^0.009
					^(b)^ <0.001
					^(f)^0.049
Calf fat area (cm^2^)	22.7 ± 10.4	24.6 ± 12.6	25.1 ± 10.1	25.4 ± 10.8	^(b)^0.001
					^(c)^ <0.001
					^(e)^0.003
					^(f)^0.000
BMI (kg/m^2^)	22.7 ± 2.8	24.8 ± 3.6	26.4 ± 3.4	23.2 ± 3.3	^(a)^0.018
					^(b)^0.012
					^(c)^0.017
					^(d)^0.012
					^(e)^0.003
					^(f)^0.003

^(a)^Significant differences between 1st and 2nd trimester; ^(b)^significant differences between 1st and 3rd trimester; ^(c)^significant differences between 2nd and 3rd trimester; ^(d)^significant differences between 1st trimester and postpartum; ^(e)^significant differences between 2nd trimester and postpartum; ^(f)^significant differences between 3rd trimester and postpartum; ns: nonsignificant.

**Table 3:** Participants' ground reaction force (GRF), joint moments, and powers, in the four collection phases.

Variables	1st trimester	2nd trimester	3rd trimester	Postpartum
3rd peak of vertical GRF (% body weight)	1.14 ± 0.04	1.13 ± 0.04	1.09 ± 0.03	1.16 ± 0.04
2nd peak of sagittal ankle joint moment (N·m/kg)	−1.36 ± 0.09	−1.34 ± 0.10	−1.28 ± 0.06	−1.34 ± 0.11
1st peak of sagittal hip joint moment (N·m/kg)	−0.61 ± 0.17	−0.53 ± 0.15	−0.48 ± 0.17	−0.62 ± 0.20
2nd peak of sagittal hip joint moment (N·m/kg)	0.77 ± 0.20	0.69 ± 0.15	0.61 ± 0.13	0.90 ± 0.23
1st peak of transverse hip joint moment (N·m/kg)	−0.26 ± 0.05	−0.32 ± 0.08	−0.30 ± 0.11	−0.26 ± 0.09
2nd peak of sagittal knee joint power (W/kg)	0.34 ± 0.30	0.35 ± 0.32	0.28 ± 0.16	0.49 ± 0.38
2nd peak of sagittal hip joint power (W/kg)	−0.75 ± 0.32	−0.67 ± 0.27	−0.61 ± 0.24	−0.99 ± 0.47
1st peak of transverse hip joint power (W/kg)	−0.31 ± 0.14	−0.67 ± 0.78	−0.41 ± 0.20	−0.35 ± 0.20

**Table 4:** Linear regression models for joints moment in the sagittal plane of motion; joints moment in the transverse plane of motion; joints power in the sagittal plane of motion; joints power in the transverse plane of motion; and ground reaction forces.

Regression models for joints moment in the sagittal plane of motion			

2nd trimester			

1st peak of hip joint moment (N·m/kg)			

Predictor variable	*B*	*β* (*p*)	adj *R* ^2^

Abdominal-gluteal ratio	−1.928	−0.665 (0.026)	0.380

3rd trimester			

2nd peak of hip joint moment (N·m/kg)			

Predictor variable	*B*	*β* (*p*)	adj *R* ^2^

Fat-free mass weight (kg)	−0.021	−0.762 (0.006)	0.534

Postpartum			

2nd peak of hip joint moment (N·m/kg)			

Predictor variable	*B*	*β* (*p*)	adj *R* ^2^

Body weight gain (kg)	0.038	0.606 (0.048)	0.296

Regression models for joints moment in transverse plane of motion			

2nd trimester			

1st peak of hip joint moment (N·m/kg)			

Predictor variable	*B*	*β* (*p*)	adj *R* ^2^

% body fat	−0.016	−0.632 (0.037)	0.333

3rd trimester			

1st peak of hip joint moment (N·m/kg)			

Predictor variable	*B*	*β* (*p*)	adj *R* ^2^

Body mass index (kg/m^2^)	−0.020	−0.606 (0.048)	0.296
Calf fat area (cm^2^)	−0.007	−0.620 (0.042)	0.316
Thigh fat area (cm^2^)	−0.003	−0.626 (0.039)	0.324

Regression models for joints power in the sagittal plane of motion			

1st trimester			

2nd peak of knee joint power (W/kg)			

Predictor variable	*B*	*β* (*p*)	adj *R* ^2^

Body weight gain (kg)	0.084	0.836 (0.010)	0.650

2nd trimester			

2nd peak of hip joint power (W/kg)			

Predictor variable	*B*	*β* (*p*)	adj *R* ^2^

Thigh fat area (cm^2^)	0.006	0.628 (0.038)	0.328

3rd Trimester			

2nd peak of hip joint power (W/kg)			

Predictor variable	*B*	*β* (*p*)	adj *R* ^2^

Fat-free mass weight (kg)	0.037	0.722 (0.012)	0.468

Postpartum			

2nd peak of hip joint power (W/kg)			

Predictor variable	*B*	*β* (*p*)	adj *R* ^2^

Fat-free mass weight (kg)	0.108	0.838 (0.001)	0.669

Regression models for joints power in the transverse plane of motion			

3rd trimester			

1st peak of hip joint power (W/kg)			

Predictor variable	*B*	*β* (*p*)	adj *R* ^2^

Body weight gain (kg)	−0.065	−0.740 (0.036)	0.473

Regression models for ground reaction forces			

3rd trimester			

3rd peak of vertical GRF (% body weight)			

Predictor variable	*B*	*β* (*p*)	adj *R* ^2^

Biiliocristal-biacromial ratio	0.476	0.679 (0.044)	0.384
